# Interaction Effects of Long-Term Air Pollution Exposure and Variants in the *GSTP1, GSTT1* and *GSTCD* Genes on Risk of Acute Myocardial Infarction and Hypertension: A Case-Control Study

**DOI:** 10.1371/journal.pone.0099043

**Published:** 2014-06-10

**Authors:** Anna Levinsson, Anna-Carin Olin, Lars Modig, Santosh Dahgam, Lena Björck, Annika Rosengren, Fredrik Nyberg

**Affiliations:** 1 Occupational and Environmental Medicine, Department of Public Health & Community Medicine, Institute of Medicine, Sahlgrenska Academy, University of Gothenburg, Gothenburg, Sweden; 2 Department of Public Health and Clinical Medicine, Occupational and Environmental Medicine, University of Umeå, Umeå, Sweden; 3 Department of Molecular and Clinical Medicine, Institute of Medicine, Sahlgrenska Academy, University of Gothenburg, Gothenburg, Sweden; 4 AstraZeneca R&D, Mölndal, Sweden; University of Western Ontario, Canada

## Abstract

**Introduction:**

Experimental and epidemiological studies have reported associations between air pollution exposure, in particular related to vehicle exhaust, and cardiovascular disease. A potential pathophysiological pathway is pollution-induced pulmonary oxidative stress, with secondary systemic inflammation. Genetic polymorphisms in genes implicated in oxidative stress, such as *GSTP1*, *GSTT1* and *GSTCD*, may contribute to determining individual susceptibility to air pollution as a promoter of coronary vulnerability.

**Aims:**

We aimed to investigate effects of long-term traffic-related air pollution exposure, as well as variants in *GSTP1*, *GSTT1* and *GSTCD*, on risk of acute myocardial infarction (AMI) and hypertension. In addition, we studied whether air pollution effects were modified by the investigated genetic variants.

**Methods:**

Genotype data at 7 single nucleotide polymorphisms (SNPs) in the *GSTP1* gene, and one in each of the *GSTT1* and *GSTCD* genes, as well as air pollution exposure estimates, were available for 119 AMI cases and 1310 randomly selected population controls. Population control individuals with systolic blood pressure ≥140 mmHg, diastolic blood pressure ≥90 mmHg or on daily antihypertensive medication were defined as hypertensive (n = 468). Individual air pollution exposure levels were modeled as annual means of NO_2_ (marker of vehicle exhaust pollutants) using central monitoring data and dispersion models, linking to participants' home addresses.

**Results:**

Air pollution was significantly associated with risk of AMI: OR 1.78 (95%CI 1.04–3.03) per 10 µg/m^3^ of long-term NO_2_ exposure. Three *GSTP1* SNPs were significantly associated with hypertension. The effect of air pollution on risk of AMI varied by genotype strata, although the suggested interaction was not significant. We saw no obvious interaction between genetic variants in the GST genes and air pollution exposure for hypertension.

**Conclusion:**

Air pollution exposure entails an increased risk of AMI, and this risk differed over genotype strata for variants in the *GSTP1*, *GSTT1* and *GSTCD* genes, albeit not statistically-significantly.

## Introduction

Exposure to air pollution has been associated with several cardiovascular (CV) outcomes such as atherosclerosis, progression of CV disease (CVD) and CV mortality. [1], [2] However, the exact biological mechanisms by which pulmonary air pollution exposure leads to cardiovascular disease outcomes are not fully understood. A proposed pathway is that pulmonary exposure induces pulmonary oxidative stress, which leads to release of pro-thrombotic and inflammatory cytokines into the blood, as well as an increased level of reactive oxygen species (ROS) in the heart. [1], [3] This enhances expression of inflammatory cytokine-genes, which in turn induces systemic inflammation and systemic oxidative stress. Inflammation furthers progress of atherosclerosis and can potentially trigger acute plaque rupture. The release of pro-thrombotic agents into the blood can also lead to clot formation and put the individual at increased risk of ischemic heart disease. Other pathways have also been suggested, e.g. translocation of particles across the pulmonary epithelium and lung-blood barrier into the cardiovascular system, which has been shown in both animals and humans. [4]

A recent review by the American Heart Association concludes that the evidence is consistent with a causal relationship between exposure to particulate matter (PM) air pollution <2.5 µm in diameter (PM_2.5_) and cardiovascular disease. [5] However, Europe had no air quality target value for PM_2.5_ until 2010 and the limit value does not enter into force until 2015, and to date longitudinal studies measuring population exposure to PM and associations with CVD are lacking. Nitrogen dioxide (NO_2_) is a gaseous air pollutant which is highly correlated with traffic intensity, and often used as indicator for vehicle exhaust in general. [1] The annual average limit for NO_2_ in the European Union has been set to 40 µg/m^3^ and has been in force since 2010. Several European studies have found an association between NO_2_ exposure and cardiovascular mortality, well below 40 µg/m^3^ [6].

Genetic polymorphisms may contribute to determining individual susceptibility to air pollution exposure as a promoter of coronary vulnerability and trigger of acute coronary events. [7] The human body can counteract the damaging effects of oxidative stress with detoxifying enzymes, and genetic variants in and surrounding the genes coding for such enzymes may determine the severity of oxidative stress. One such detoxification enzyme is the dimeric Glutathione S-Transferase (GST), whose specific role is to protect against the products of oxidative stress. [8] The *GST* gene family in humans is complex, consisting of 16 genes in six subgroups including *GSTT1, GSTM1* and *GSTCD*. [9] *GST* gene expression is induced by compounds linked to chemical stress and carcinogenesis. [10], [11]

Variants in the *GSTP1* gene have been shown to modify the effect of air pollution exposure on lung function [12], and a few studies have also studied *GSTP1* variants regarding interaction with air pollution exposure in CVD outcomes such as blood pressure, but to date no significant interactions have been reported. [13], [14] A genome wide association study (GWAS) identified an association between decreased pulmonary function and SNPs in *GSTCD* (glutathione S-transferase, C-terminal domain containing), a result which was supported by an extended meta-analysis of several GWASs. [10], [15] So far, no associations between *GSTCD* and cardiovascular disease have been reported. The polymorphism in the *GSTT1* gene is caused by a gene deletion, resulting in almost no enzymatic activity in individuals with the null genotype; hypothetically putting the individual at increased risk of oxidative stress and downstream effects e.g. atherosclerosis and coronary heart disease. [11], [16]

Hence, a probable pathway between air pollution and cardiovascular effects is oxidative stress, but the relationship between variants in antioxidant genes and cardiovascular disease as well as potential interaction between such genetic variants and air pollution exposure in cardiovascular disease is incompletely explored. We hypothesized that there is an interaction between air pollution exposure and genetic variants in the GST genes on risk of acute myocardial infarction (AMI) and thus we aimed to investigate, in a population-based study, a) the association of long-term traffic-related air pollution exposure with risk of acute myocardial infarction (AMI) and hypertension, b) the associations of genetic variants in *GSTP1*, *GSTCD* and *GSTT1* with risk of AMI and hypertension, and c) if these genetic variants modify the associations between air pollution exposure and the two outcomes.

## Methods

### Ethics statement

The study was approved by the local ethical committee and all participants provided written informed consent.

### Study population and data collection

The INTERGENE/ADONIX (INTERplay between GENEtic susceptibility and environmental factors for the risk of chronic diseases in West Sweden/ADult-Onset asthma and exhaled NItric oXide) study has been described in detail elsewhere. [17], [18] In brief, from April 2001 until December 2004, INTERGENE/ADONIX recruited coronary heart disease (CHD) cases and a population control cohort from the greater Gothenburg area in Sweden. The population control cohort consisted of 3614 randomly selected individuals aged 25–75 years at time of selection. As CHD cases, the study included consecutive patients admitted to wards at 3 locations (Östra, Mölndal and Sahlgrenska) of the Sahlgrenska university hospital, Gothenburg, Sweden or outpatients with significant coronary lesions identified from coronary angiograms. Altogether, the INTERGENE/ADONIX study included 618 patients (73.4% men and 26.6% women) with CHD, 295 with a first episode of acute myocardial infarction (AMI) or unstable angina pectoris, and the rest with chronic CHD, defined as either prior AMI or positive angiogram. The upper age limit was 75 years. 192 patients were individuals presenting with first-time acute myocardial infarction (AMI) and included as cases in the present study.

Study participants received questionnaires and were invited to a medical examination. During the medical examination, height and body weight was measured to the nearest 1 cm and 0.1 kg with the subjects in light clothing and without shoes. Body mass index (BMI) was calculated from weight (kg) and height (m) using the formula BMI = weight/height^2^. Blood pressure measurement was carried out in a sitting position after 5 minutes rest, using a validated blood pressure device (Omron 711 Automatic IS; Omron Healthcare Inc., Vernon Hills, IL). The blood pressure was measured twice and then the mean of the two measurements was used. Blood samples were collected after 4 hours of fasting for immediate serum lipid (total cholesterol, HDL cholesterol and triglycerides) analysis and storage for DNA extraction.

Based on the questionnaire, the individuals' level of education was classed as primary if highest achieved education was elementary school, lower secondary school, vocational school or girls' school. Secondary meant that the individual indicated having attended upper secondary school or high school and tertiary signified having attended university or college.

Individuals with a systolic blood pressure (SBP) ≥140 mmHg, or diastolic blood pressure (DBP) ≥90 mmHg or who were taking daily antihypertensive medication were defined as ‘hypertensive’. Individuals of non-European birth (5%) were excluded from the current genetic analyses. Of those reporting European origin and included, 90% reported being of Swedish origin.

### Air pollution exposure

Modeled annual average levels of NO_2_ outside each participant's baseline home address were used for exposure assessment. Each participant's home address was translated into geographical coordinates and combined with modeled levels of NO_2_ in a geographical information system (GIS). The dispersion model, which is hosted by the local authorities, contains both emission data and meteorological information and has been previously validated against actual measurements, showing good agreement.[19] The main output from the model is NO_x_ values with high spatial resolution (20×20 meters), which were then converted to estimated NO_2_ using local empirical relationships. Due to the availability of concentration grids, the calculated exposure levels represented the years 2006 and 2007 and not the exact years of inclusion (2001–2004). For individuals with air pollution data for both years, we used the 2007 value because the geographical area covered was increased from 2006. For individuals with exposure data from only one year, this value was used. Correlation between values for individuals with values from both years was 0.98 for NO_2_. This high degree of stability over years indicates that 2007 is a good indicator also for the long-term spatial distribution of exposure levels during 2001–2004.

### SNP selection and genotyping

7 SNPs from the *GSTP1* gene and one SNP from each of the *GSTT1* and *GSTCD* genes were selected from the literature. SNPs were genotyped using a Sequenom MassARRAY platform (Sequenom San Diego, CA, USA) or a competitive allele-specific PCR system KASPar (KBioscience, Hoddesdon Herts, GB), all with a call rate ≥90%. SNPs with a Hardy-Weinberg Equilibrium (HWE) p-value ≤0.05 were excluded, as were individuals missing results for more than two out of seven *GSTP1* SNPs. The *GSTT1* SNP rs2266637 (as a marker of the null genotype) was genotyped twice and the resulting concordant pairs of null/non-null genotype individuals from the two assays were used for analysis.

### Statistical analysis

Methods are analogous for AMI and hypertension, except that for hypertension the analysis was restricted to the population control sample. All analyses used logistic regression models adjusted for age, age squared and gender. Potential selection bias for cases and controls due to geographical clustering of cases' home addresses in areas closer to the two source hospitals was accounted for by adjusting for residential area based on the postal code, since within these smaller areas the distribution of cases and controls was representative. Consequently, all analyses concerning air pollution were also adjusted for residential area and educational level (main effects and interaction analyses), while for all analyses involving genotypes, BMI was included in the model (main effects and interaction analyses).

First, effects of NO_2_ (as a marker for vehicle exhaust pollutants) on risk of AMI or hypertension were analyzed separately. Thereafter, effects of genetic variants on risk of AMI and hypertension were studied. For *GSTP1*, each of the 7 SNPs was analyzed coded to the dominant genetic model (0 for two copies of the major allele, 1 for heterogeneous genotype or two copies of the minor allele). [20] For this gene, only the SNP or SNPs with the strongest effects on AMI or hypertension were studied for interaction with air pollution exposure on risk of each respective outcome. For *GSTCD*, a single variant (rs10516526) was studied, coded to the dominant genetic model. The *GSTT1* null genotype was studied using the two genotypes captured by the SNP rs2266637. Finally, interaction between air pollution and genetic variants was investigated by estimating effects of air pollution in analyses stratified by genotype in a common regression model. For this model, we also assessed whether smoking might modify the interaction between air pollution and genetic variants on AMI.

## Results

In total, 1429 individuals had both valid genotype and air pollution exposure data and could be included in the analyses; 119 of the 192 first-time AMI cases, and 1310 of the 3614 control individuals. The reduction in numbers was mainly due to the geographical area covered by the dispersion model (for details, see Figures S1a–b). As expected, AMI cases were significantly different from controls on several characteristics; in particular, cases were more often male and older than controls (Table 1). In the population control cohort, 468 (36%) individuals were classified as hypertensive. Over 90% of AMI cases were taking daily medication for high cholesterol, but only 17% of hypertensive controls and 2% of normotensive controls; most likely this explains the lower mean cholesterol levels among cases compared to controls. Mean annual value of NO_2_ exposure across all subjects was 15.6 with standard deviation 6.1.

**Table 1:** Demographic characteristics of the 1429 individuals in the study population.

		AMI cases	Population controls		
			*Hypertensive*	*Non-hypertensive*	
		n = 119	n = 468	n = 842	
Characteristic		Mean (SD)	Mean (SD)	Mean (SD)	p-value
Age, years		60.7 (8.35)	59.5 (11.7)	44.0 (12.7)	0.0001*
BMI, kg/m^2^		27.6 (3.80)	27.6 (4.43)	24.8 (3.73)	0.0001*
LDL cholesterol, mmol/l		2.38 (0.91)	3.51 (0.99)	3.13 (0.99)	0.0001*
HDL cholesterol, mmol/l		1.28 (0.36)	1.61 (0.48)	1.67 (0.45)	0.0001*
Total cholesterol, mmol/l		4.47 (1.12)	5.82 (1.08)	5.34 (1.10)	0.0001*
SBP, mmHg		133 (21.9)	149 (18.9)	116 (11.6)	0.01*
DBP, mmHg		81 (11.1)	89 (10.2)	76 (7.20)	0.94*
NO_2_, µg/m^3^		14.9 (5.75)	14.9 (5.93)	16.1 (6.16)	0.20*
		**N (%)**	**N (%)**	**N (%)**	
Age	≤34	0 (0%)	16 (3.4%)	232 (27.6%)	<0.0001∧
35–44		3 (2.5%)	47 (10.0%)	248 (29.5%)	
45–54		26 (21.8%)	74 (15.8%)	177 (21.0%)	
55–64		46 (38.7%)	136 (29.1%)	118 (14.0%)	
≥65		44 (37.0%)	195 (41.7%)	67 (7.9%)	
					
Women		36 (30.3%)	244 (52.1%)	481 (57.1%)	0.0001∼
Smoking status §	Never	29 (24.8%)	204 (43.9%)	429 (51.3%)	<0.0001∧
	Former	73 (62.4%)	171 (36.8%)	239 (28.6%)	
	Current	15 (12.8%)	90 (19.4%)	169 (20.2%)	
Diabetes		15 (12.6%)	35 (7.49%)	12 (1.43%)	0.04∼
Daily lipid-lowering medication		94 (91.3%)	57 (16.9%)	14 (2.40%)	0.0001∼
Daily blood pressure-lowering medication		71 (74.7%)	150 (39.9%)	0 (0%)	0.0001∼
				
Level of education	Primary	91 (76.5%)	289 (61.8%)	241 (28.6%)	<0.0001∧
	Secondary	12 (10.1%)	68 (14.5%)	206 (24.5%)	
	Tertiary	16 (13.5%)	103 (22.0%)	388 (46.1%)	

*p-values refer to test of equality of means between AMI cases and all controls.

∧ p-values refer to chi^2^ test between AMI cases and all controls.

∼ p-values refer to test of proportions between AMI cases and all controls.

§ Smoking status: smoking of cigarettes, pipe, cigar or cigarillos.

AMI: acute myocardial infarction; SD: standard deviation; BMI: Body Mass Index; LDL: low-density lipoprotein; HDL: high-density lipoprotein; SBP: systolic blood pressure; DBP: diastolic blood pressure.

### Main effects of air pollution exposure and genetic variants on risk of AMI and hypertension

Air pollution was significantly associated with increased risk of AMI; odds ratio (OR) 1.78 (95% confidence interval (CI) 1.04–3.03) per 10 µg/m^3^ of NO_2_ (Table 2).

**Table 2:** Effect of long-term traffic-related air pollution exposure (using annual mean of NO_2_ as exposure indicator) on risk of acute myocardial infarction (AMI) and hypertension.

Effect per 10 µg/m^3^ of NO_2_					
AMI			Hypertension		
OR	95% CI	p-value	OR	95% CI	p-value
1.78	1.04–3.03	0.03	0.82	0.57–1.19	0.30

*Model covariates are sex, age, age squared, level of education and residential area.

AMI: acute myocardial infarction, OR: odds ratio, CI: confidence interval.

None of the seven *GSTP1* SNPs showed a significant association with AMI; the most strongly associated with AMI was rs596603, with OR 0.77 (95% CI 0.51–1.16) (Table 3). In contrast, 5 of the 7 *GSTP1* SNPs had a p-value≤0.05 for hypertension; the 3 strongest of these (rs1871042, rs749174 and rs762803) remained significant after Bonferroni-correction (p-value≤0.007; Table 3). Neither the *GSTCD* SNP rs10516526 nor the *GSTT1* null genotype showed significant main effect results for AMI or hypertension. All SNPs in Table 3 were investigated further in the interaction analyses.

**Table 3:** Effects of the most significant variants in *GSTP1*, the *GSTCD* SNP rs10516526 (dominant genetic model) and the *GSTT1* SNP rs2266637 (null genotype) on risk of acute myocardial infarction (AMI) and hypertension.

		Genotype frequencies				Effect estimates and precision*		
			Cases	Controls	Genetic			
Gene: SNP	Outcome		no. (%)∧	no. (%)∧	Model	OR	95% CI	p-value
*GSTP1:*	AMI	TT	20 (17%)	245 (19%)	(TT+GT) vs.	0.77	0.51–1.16	0.21
rs596603		GT	53 (45%)	659 (51%)	GG			
		GG	45 (38%)	398 (30%)				
*GSTP1:*	Hypertension	TT	50 (10%)	100 (12%)	(TT+TC) vs.	0.66	0.50–0.87	0.003
rs1871042		TC	201 (44%)	406 (49%)	CC			
		CC	211 (46%)	319 (39%)				
*GSTP1:*	Hypertension	AA	51 (11%)	102 (12%)	(AA+AG) vs.	0.66	0.50–0.88	0.004
rs749174		AG	202 (43%)	413 (49%)	GG			
		GG	213 (46%)	326 (39%)				
*GSTP1:*	Hypertension	AA	85 (19%)	148 (18%)	(AA+CA) vs.	0.66	0.49–0.89	0.006
rs762803		CA	218 (47%)	442 (54%)	CC			
		CC	156 (34%)	230 (28%)				
*GSTCD*:	AMI	GG	0	10 (1%)	(GG+AG) vs.	0.69	0.34–1.38	0.29
rs10516526		AG	10 (9%)	128 (10%)	AA			
		AA	108 (91%)	1152 (89%)				
	Hypertension	GG	4 (1%)	6 (1%)	(GG+AG) vs.	0.87	0.55–1.36	0.53
		AG	50 (11%)	78 (10%)	AA			
		AA	407 (88%)	745 (89%)				
*GSTT1:*	AMI	Null	11 (10%)	168 (14%)	Null vs.	0.65	0.33–1.27	0.20
rs2266637		Non-null	101 (90%)	1053 (86%)	Non-null			
	Hypertension	Null	58 (13%)	110 (14%)	Null vs.	0.88	0.59–1.33	0.55
		Non-null	382 (87%)	671 (86%)	Non-null			

*Adjusted for age, age squared, BMI and sex.

∧ Percent of non-missing.

AMI: acute myocardial infarction, OR: odds ratio, CI: confidence interval, BMI: body mass index.

### Interaction analysis

We saw no significant overall interaction between any of the genetic variants in the GST genes and air pollution exposure for AMI or hypertension. However, for all SNPs tested in the case of AMI (GSTP1:rs596603, GSTCD:rs10516526 and GSTT1:rs2266637), one genotype stratum has an effect estimate for air pollution exposure on risk of AMI that is significant and almost twice that of the other stratum, suggesting that the genotype may affect the adverse effect of NO_2_ exposure. (Table 4)

**Table 4:** Interaction between long term air pollution (using annual mean of NO_2_ as exposure indicator) and SNPs in *GST* genes on risk of acute myocardial infarction and hypertension.

			Effects per 10 µg/m^3^ of NO_2_ *			
*Gene*: SNP	Outcome	Genotype	OR	95% CI	p-value	Interaction p-value
*GSTP1*: rs596603	AMI	TT+GT	2.12	1.09–4.10	0.03	0.27
		GG	1.40	0.73–2.68	0.31	
						
*GSTP1:* rs1871042	Hypertension	TT+TC	0.79	0.50–1.24	0.31	0.62
		CC	0.89	0.57–1.39	0.61	
						
*GSTP1:* rs749174	Hypertension	AA+AG	0.78	0.50–1.22	0.28	0.57
		GG	0.90	0.58–1.39	0.63	
						
*GSTP1*: rs762803	Hypertension	AA+CA	0.96	0.63–1.45	0.84	0.36
		CC	0.76	0.46–1.25	0.28	
*GSTT1*: rs2266637	AMI	Null	1.40	0.33–5.96	0.65	0.60
		Non-null	2.02	1.13–3.60	0.02	
	Hypertension	Null	0.84	0.38–1.85	0.66	0.89
		Non-null	0.89	0.60–1.31	0.54	
*GSTCD*: rs10516526	AMI	GG+AG	1.01	0.28–3.73	0.98	0.23
		AA	2.25	1.25–4.06	0.007	
	Hypertension	GG+AG	0.87	0.40–1.88	0.72	0.93
		AA	0.90	0.60–1.35	0.61	

*Adjusted for age, age squared, sex, BMI, level of education and residential area.

OR: odds ratio; CI: confidence interval, AMI: acute myocardial infarction.

## Discussion

This study's main findings are an association between indicators of long-term vehicle exhaust exposure and acute myocardial infarction, and an effect for several variants in the *GSTP1* gene on risk of hypertension. Further, this study of interaction between long-term vehicle exhaust exposure and genetic susceptibility in CVD further suggests that cardiovascular response to air pollution exposure may be modified by genetic variants in the GST genes, although the power for interaction analysis was limited.

Which constituents in the heterogeneous traffic-related air pollution mix are actually responsible for the adverse effect is unknown, and for comparability with other European studies, we have used NO_2_ as a marker for vehicle air pollution exposure in the association analyses. The finding of an association between long-term traffic-related air pollution exposure and AMI is consistent with some earlier studies. [5], [7], [21] Several European cohort studies have found significant associations between NO_2_ and cardiovascular mortality, with estimates ranging from 1.05 to 1.39 per 10 µg/m^3^.[6] These estimates are lower than those found in the present study, but considering the important difference in endpoint this may be expected. Rosenlund et al [22] found a suggested association between long-term average air pollution and fatal MI (OR 1.51 95% CI 0.96–2.16 for 5^th^ to 95^th^ percentile difference in 30-year average exposure to NO_2_) in Stockholm, but none for non-fatal MI, which gives a null effect overall (OR 0.99 95% CI 0.76–1.30 for 5^th^ to 95^th^ percentile difference in 30-year average exposure to NO_2_).

While American studies have focused more on between-city studies, European studies have mostly investigated within-city contrasts. This makes the comparability with American studies poor. It has been suggested that within-city contrasts are more strongly associated with cardiovascular events than between-city differences.[23] A recent Danish study [24] also using modeled NO_2_ exposure at residential addresses found that NO_2_ at the baseline address showed weaker associations than when using all addresses from 1 January 1971 until death or 31 December 2009 – presumably due to non-differential misclassification of exposure when using one address as a proxy for longer-term exposure. Since we have used NO_2_ data from the baseline address only, our estimate may similarly be more likely to underestimate the association than overestimate it.

Due to the properties of the gases and particles constituting vehicle exhaust, inhalation of the exhaust may cause oxidative stress [7], [25], [26], [27], and this is potentially the mechanism for the association between such air pollution exposure and cardiovascular disease, especially when antioxidant and reductive response is weakened. This connection of inhaled air pollution to CVD is sometimes called the indirect pathway. The *GSTT1, GSTCD* and *GSTP1* genes code for metabolizing enzymes that are involved in counteracting the effects of oxidative stress. [8] The three GSTP1 SNPs which showed a main effect on risk of hypertension are in high linkage disequilibrium with each other (Figure S2), indicating that they may be markers of the same effect. To our knowledge, no associations between hypertension and these SNPs have been published to date.

In addition to the *GSTP1* SNPs' association with hypertension, our results also show a significant adverse effect of air pollution exposure on AMI for the subjects with AA-genotype in *GSTCD* SNP rs10516526, OR 2.25 (95% CI 1.25–4.06) per 10 µg/m^3^ of NO_2_, and a near null effect estimate for subjects with GA or GG genotypes combined (OR 1.01, 95% CI 0.28–3.73). To our knowledge, nothing has previously been reported regarding an association of variants in the *GSTCD* gene with cardiovascular outcomes. What has been reported is a positive association between the G-allele in the same *GSTCD* SNP and forced expiratory volume in the first second (FEV_1_). [10], [15] Thus it seems that the G-allele may be beneficial both for lung function and protection against cardiovascular disease.

The null polymorphism of the *GSTT1* gene is present in approximately 20% of Caucasians and has been associated with lower levels of high-density lipoprotein (HDL) cholesterol, elevated triglyceride-HDL ratio and hypertriglyceridemia, all risk factors for CVD. [11] A meta-analysis of 19 studies (total of 8020 coronary heart disease (CHD) cases and 11501 controls) gave an overall relative risk of CHD of 1.26 (95% confidence interval (CI): 0.90–1.75) for the *GSTT1* null polymorphism. [28] This effect was modified by smoking; for smoking null genotype individuals, there was a high and significantly increased risk of CHD (OR 3.29, 95% CI: 1.46–7.26). In another study of patients suspected of coronary artery disease who had undergone coronary angiography, however, no association between the *GSTT1* null genotype and coronary atherosclerosis (defined as >20% obstructive lesion) was seen. [29] In the present study, less obvious air pollution effect variation by genotype was suggested for GSTP1 rs596603 and the GSTT1 null variant, where air pollution exposure was associated with a higher and nominally significant risk of AMI in subjects with GT or TT genotype in GSTP1 rs596603 vs GG (OR 2.12, 95% CI 1.09–4.10 vs OR 1.40, 95% CIs 0.73–2.68), as well as for individuals carrying the non-null genotype of *GSTT1*, i.e. the presence of a coding gene, compared to subjects with the null genotype (OR 2.02, 95% CI 1.13–3.60 vs OR 1.40, 95% CI 0.33–5.96). The latter result is somewhat counterintuitive; however, the system which counteracts the effects of oxidative stress is complex, and the balance between involved enzymes remains to be explored. Neither of these suggested interactions were however statistically significant, reflecting the limited power of interaction testing.

The INTERGENE/ADONIX study is a well characterized, population-based study with high-quality genotyping data. Unfortunately, calculated long-term air pollution data were not available for the entire geographical study area, thus excluding many individuals with otherwise valid data from the present analysis. Modeling air pollution levels for a larger geographical area would enable inclusion of more of the participants and increased power, but such models remain to be developed.

Starting with a list of potential covariates and confounders from literature, we assessed confounding for each variable. Smoking was also investigated for potential effect modification of the gene-environment interaction, but none was found. Residential area and educational level are important for reducing any geographical or socio-economic selection bias. BMI seemed to vary by genotype and was thus included in the genotype analyses.

A potential complication from adjusting for residential area in the air pollution analyses is some loss of exposure contrast, since this means that effects are essentially estimated within each residential area and then pooled across areas. However, the adjustment addresses potential confounding from area-related subject characteristics (e.g. socio-economic status and lifestyle characteristics), or geographical selection bias. In addition, sufficient contrast generally remains even within smaller geographical areas, as indicated also in the current analysis by the significance of the association between air pollution exposure and AMI that we observed.

## Conclusions

In addition to giving insight into the specific pathways of disease pathology, results from gene-environment interaction studies can provide additional possibilities of identifying subjects at particularly high risk (i.e. those with high-risk genotypes ***and*** high-risk environment), and potentially to give them targeted prevention, health advice and care. This study shows significant association of vehicle air pollution exposure with risk of AMI; effects of variants in the GSTP1 gene on risk of hypertension, and suggests interactions between variants in the GSTP1, GSTT1 and GSTCD genes and vehicle air pollution exposure on risk of AMI.

## Supporting Information

Figure S1 a) Geographical area covered by the dispersion model used to calculate annual average NO_2_ exposure in 2006, b) geographical area covered by the dispersion model used to calculate annual average NO_2_ exposure in 2007.
(GIF)

Figure S2 **Linkage disequilibrium (LD) plot for** ***GSTP1*** **gene SNPs, with values in each cell representing R^2^ between pairs of SNPs and coloring representing D′.**
(GIF)
