# Supplementary figures and images for: Interaction Effects of Long-Term Air Pollution Exposure and Variants in the GSTP1, GSTT1 and GSTCD Genes on Risk of Acute Myocardial Infarction and Hypertension: A Case-Control Study
Source: PLoS One. 2014 Jun 10;9(6):e99043. doi: 10.1371/journal.pone.0099043 (PMC4051658; doi:10.1371/journal.pone.0099043)

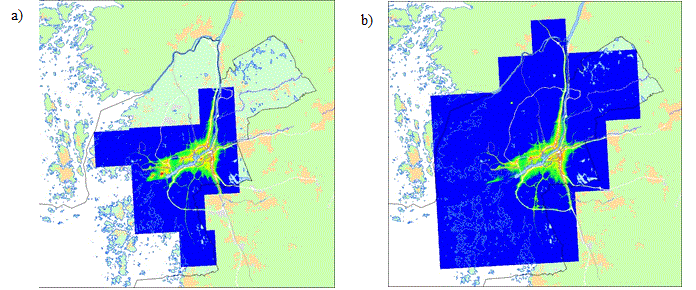

Supplement: Figure S1 — a) Geographical area covered by the dispersion model used to calculate annual average NO2 exposure in 2006, b) geographical area covered by the dispersion model used to calculate annual average NO2 exposure in 2007. (GIF) [file pone.0099043.s001.gif]

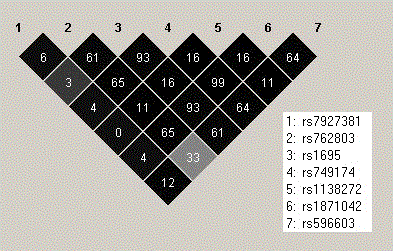

Supplement: Figure S2 — Linkage disequilibrium (LD) plot for GSTP1 gene SNPs, with values in each cell representing R2 between pairs of SNPs and coloring representing D′. (GIF) [file pone.0099043.s002.gif]
